# Reliability Analysis of HHV Prediction Models for Organic Materials Using Bond Dissociation Energies

**DOI:** 10.3390/polym15193862

**Published:** 2023-09-22

**Authors:** Junjun Tao, Longwei Pan, Jiajie Yao, Longfei Liu, Qiang Chen

**Affiliations:** School of Materials Engineering, Changshu Institute of Technology, Changshu 215500, China; jjtao@cslg.edu.cn (J.T.); longweipan@cslg.edu.cn (L.P.); yaojj@cslg.edu.cn (J.Y.)

**Keywords:** reliability analysis, heating value, bond dissociation energy, oxygen consumption

## Abstract

The purpose of this study is to analyze the reliability of predictive models for higher heating values related to organic materials. A theoretical model was developed, which utilizes bond dissociation energies (BDEs) to establish correlations between elemental composition and calorific values. Our analysis indicates that the energy contribution of one mole of hydrogen atoms is approximately equal to −144.4 kJ mol^−1^. Further investigation reveals significant variations in the bond dissociation energies of carbon atoms within organic compounds, resulting in a range of energy outputs from −414.30 to −275.34 kJ mol^−1^ per mole of carbon atoms. The presence of oxygen atoms in organic compounds has a negative impact on the magnitude of combustion heat, with values ranging from 131.1 to 207.17 kJ mol^−1^. The combustion mechanism imposes certain constraints, leading to the equation HHV*_g_* = −31.34·[C] − 144.44·[H] + 10.57·[O] for organic compounds. Based on the parameter sensitivity analysis, the coefficient associated with carbon mass fraction exhibits a significantly greater impact on result prediction accuracy, demonstrating a sensitivity value of 92.65%. The results of further analysis indicate that empirical correlations involving the mass fractions of the elements N and S in lignocellulosic materials may be prone to over-fitting, with sensitivity indices of 1.59% and 0.016%, respectively.

## 1. Introduction

The calorific values of organic materials have essentially been an important parameter in material flammability assessment and fire dynamic simulation [1,2,3]. Based on large amount of experimental data, researchers found that the heat released by burning organic compounds would be approximately 13.1 MJ per kilogram of oxygen consumed [4,5]. By the utilization of bond dissociation energies (BDE), Yao and Wang [6] demonstrated that C-H, C-C, and C=C bonds positively affect the calorific values, whereas O-H, C-N, C-O, and C=O bonds have the opposite effect. Based on the statistical results of calorific data of 1087 fuels, Merckel et al. [5] found that the heating value was much more sensitive to oxygen content than carbon and hydrogen content of an organic material. Clearly, calorific values have a strong dependency on the element constitution of combustible organics.

Given the complicity in performing a test and the associated costs, many studies have established large numbers of regression models of higher heating values (HHVs) with chemical composition using a mathematical fitting method [6,7,8,9,10]. In 1997, Demirbas [11] firstly reported a correlation of the heating value with the element content of lignocellulosic materials. Merckel et al. [5] proposed an exponential model of higher heating value (HHV) with the mass fraction of oxygen consumed by combustion. Dashti et al. [12] employed regression models for HHV prediction of municipal solid waste based on ultimate analysis. However, regression analysis is a statistical process and takes no account of the essence of combustion reactions. As a result, several correlations reported in the literature are indeed against the basic principle that the increase in O content would lead to a decrease in the HHV [13,14].

Furthermore, there are always no valid constraint conditions for model parameters in the fitting process, and the existing models usually have different equation forms [12,15,16,17,18]. Additionally, the variables in existing models are usually not independent where their sum was equal to 1. This results in the uncertainty of model parameters in fitting formulae, and the reliability and robustness of regression models remain to be discussed [12,16,17]. The occurrence of over-fitting phenomena may arise in order to achieve optimal fitting results, which has led to doubts about the applicability of empirical models. In light of extensive test data for various biomass fuels, Yin [15] examined the applicability and reasonability of various empirical correlations proposed in the literature and declared significant differences in the accuracy of predicting results.

The aim of this study is to conduct a reliability analysis for predictive models of higher heating values pertaining to organic materials. A theoretical model was developed, employing bond dissociation energies to establish correlations between elemental composition and calorific values. By quantifying the energy output per mole of elemental atoms, uncertainties associated with parameters within HHV prediction models can be determined. The new fitting results were subsequently compared with the existing mathematical models through parameter sensitivity analysis. The theoretical explanation for the relationship between heating values and oxygen consumption has been provided on a basis. Then, the theoretical model has been further expanded to incorporate the influence of N and S elements.

## 2. Model Development

The combustion of organic materials with the genetic formula C*_c_*H*_h_*O*_o_* can generally be described using the following reaction

(1) $$C_{c}H_{h}O_{o}+\nu_{O_{2}}O_{2}\;\to \;\nu_{CO_{2}}CO_{2}+\;\nu_{H_{2}O}H_{2}O$$

where $\nu_{O_{2}}$ = *c* + *h*/4 − *o*/2, $\nu_{CO_{2}}$ = −*c*, and $\nu_{H_{2}O}$ = −*h*/2. The heat of a chemical reaction can be determined by considering the energies involved in bond breaking and bond making. The reaction heat of an exothermic reaction is usually defined as a negative value. Then, the bond dissociation energy (BDE) represents the amount of energy needed to break a bond and is expressed as a positive value. Thus, the products coefficients in Equation (1) are negative and represent that the formation process of combustion products is exothermic. Then, the combustion heat released by reaction (1) can then be accurately evaluated as the difference between the sum of bond dissociation energies (BDEs) of reactants and products [4,6].

(2) $$HHVm\approx \overset{n}{\underset{i}{\sum }}\nu_{i}H_{rnx,i}=\overset{n}{\underset{i}{\sum }}\left(\nu_{i}\underset{m}{\sum }D_{i,m}\right)$$

where HHV*_m_* refers to the higher heating value on a molar basis, at standard conditions (i.e., 100 kPa and 25 °C). H*_rnx,i_* represents total bond dissociation energies of a substance *i*, whereas *D_i,m._* signifies the BDE value of the chemical bond *m* with respect to component *i*. The value of H*_rnx,_*_H2O_ should include the molar heat of vaporization for water (40.7 kJ mol^−1^).

The BDE values are always endothermic and have a positive enthalpy value, as shown in Table 1. Significantly, the values of H*_rnx,_*_O2_, H*_rnx,C_*_O2_, and H*_rnx,_*_H2O_ remain constant, with the results of 498.0 kJ mol^−1^, 1607.0 kJ mol^−1^, and 969.8 kJ mol^−1^, respectively. Taking CH_4_ as an example, the total BDEs of CH_4_ are approximately 1657.2 kJ mol^−1^ by adding the average BDE values of four C-H bonds, i.e., $\bar{D}\left(H-C\right)\times 4$. Then, the evaluation of Equation (2) yields −892.8 kJ mol^−1^ for the combustion heat of CH_4_, close to its actual value of −890.53 kJ mol^−1^.

An empirical formula for the calculation to predict BDE values has been proposed by Tiggelen et al. [21] as follows:

(3) $$D\left(A-B\right)\approx \frac{1}{\alpha_{m}}\left[\alpha_{A}D_{A}+\alpha_{B}D_{B}\right]$$

where *α*_A_ and *α*_B_ are constants characterizing radicals A and B. The value *α*_H_ for the H atom is definitely equal to unity, that is, *α*_H_ = 1. *α_m_* stands for the smaller of the latter, i.e., min(*α*_A_ *α*_B_). Then, the typical values of *D*_H_, *D*_C_, and *D*_O_ were, respectively, 215.95 kJ mol^−1^, 173.68 kJ mol^−1^, 77.32 kJ mol^−1^, and *α*_C_ = 1.20, *α*_O_ = 2.95 [21]. The reliability of Equation (3) has been thoroughly discussed and demonstrated through a comparison between the calculated and experimental values of bond dissociation energy, as reported by Tiggelen et al. in 1965 [21]. The discrepancies between calculated and experimental values are within a range of 8.37~12.56 kJ mol^−1^, which can be considered negligible compared to the total value H*_rxn_*(C*_c_*H*_h_*O*_o_*) of organic materials.

Element C typically forms four electron-pair bonds, while element O forms two and element H only one. It is hypothesized here that a chemical bond can be simplified as either a single bond or a combination of such bonds. Then, in light of the empirical formula Equation (3), the total bond dissociation energies of an organic matter C*_c_*H*_h_*O*_o_* can be determined using

(4) $$H_{rnx}\left(C_{c}H_{h}O_{o}\right)=\overset{4c}{\underset{R-C}{\sum }}\frac{\alpha_{C}}{\alpha_{C,m}}D_{C}+\overset{h}{\underset{R^{\prime }-H}{\sum }}\frac{\alpha_{H}}{\alpha_{H,m}}D_{H}+\overset{2o}{\underset{R^{\prime \prime }-O}{\sum }}\frac{\alpha_{O}}{\alpha_{O,m}}D_{O}$$

where the symbol $\overset{4c}{\underset{R-C}{\sum }}$ refers to the summation for all the chemical bonds with atom C, and the number of bond R−C is 4*c*. Similar definitions hold for another two accumulative symbols in Equation (4). *α*_C,*m*_ stands for the smaller of the latter, i.e., min(*α*_C_, *α*_R_), for C bonds in organic matter. For the organic compounds with established chemical structures, the carbon bonds have been simplified to C-C, C-H, and C-O, with corresponding bond numbers being determined through statistical analysis. For example, in the case of C-O bonds, *α*_C,*m*_ can be expressed as min(*α*_C_*,α*_O_*)*, with a value of 1.2. Similar connotations are attributed to other symbols. The first term on the right-hand side of Equation (4) can be determined by aggregating the outcomes. Analogous procedures are executed for the bonds involving oxygen and hydrogen.

For technical reasons, we rewrite Equation (4) as

(5) $$H_{rnx}\left(C_{c}H_{h}O_{o}\right)=4c\cdot H_{rnx,\;C}+h\cdot H_{rnx,H}+2o\cdot H_{rnx,O}$$

where H*_rnx,_*_C_ is the average of α_C_D_C_/α_C,*m*_ for element C in an organic compounds, and similar definitions apply to H*_rnx,_*_H_ and H*_rnx,_*_O_. Based on the assumption proposed above, the values of H*_rnx,_*_C_, H*_rnx,_*_H,_ and H*_rnx,_*_O_ can be determined with the numbers of single bonds. By applying the rearrangement of Equation (5) to Equation (2), an analytical model for estimating combustion heat can be derived as

(6) $${\mathrm{HHV}}_{m}\approx \varphi_{C}\cdot c+\varphi_{H}\cdot h+\varphi_{O}\cdot o$$

(7) $$\varphi_{C}=4\cdot H_{rnx,C}+H_{rnx,\;O_{2}}-H_{rnx,\;CO_{2}}$$

(8) $$\varphi_{H}=H_{rnx,H}+\frac{H_{rnx,\;O_{2}}}{4}-\frac{H_{rnx,H_{2}O}}{2}$$

(9) $$\varphi_{O}=2\cdot H_{rnx,O}-\frac{H_{rnx,O_{2}}}{2}$$

Formulas (6)–(9) describe the relationship between higher heating value and atom contents from the prescriptive of bond dissociation energies. The energy contribution of each element atom can be accurately calculated through further analysis, thereby enabling the determination of the reliability of traditional fitting equations.

## 3. Model Validation

The application of derived theoretical models has been validated by comparing the predicted HHV*_m_* values calculated using Equations (6)–(9) and theoretical values using 154 species of organic molecules with specific chemical structures [22]. The theoretical values of combustion heats are calculated based on the standard molar enthalpies of formation as documented in the scientific literature. This method has been widely accepted and yields only minor discrepancies with the experimental values. The reliability of the verification results can be ensured with a sufficient number of data samples, and errors caused by human factors can be eliminated through the use of a unified data source.

**Figure 1:**
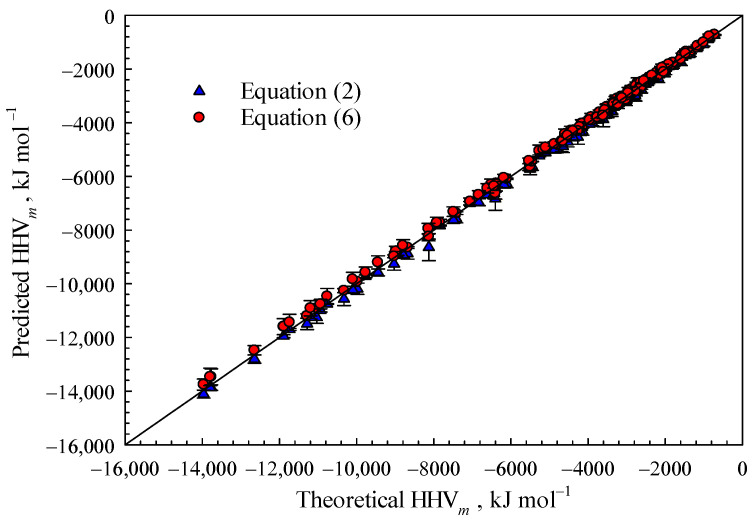
Comparison between the HHVs predicted using Equations (2) and (6) with theoretical values.

For organic molecules with well-defined chemical structures, the species and quantities of chemical bonds can be enumerated, subsequently enabling the calculation of H*_rnx_*_,C_, H*_rnx_*_,H_, and H*_rnx_*_,O_ values. The HHV*_m_* values and coefficients *φ*_C_, *φ*_H_, and *φ*_O_ are determined using Equations (6)–(9) as the basis. Both calculating formulae Equations (2) and (6) were employed as illustrated in Figure 1. The organic molecules comprise aliphatic, alicyclic and aromatic hydrocarbons as well as aliphatic alcohols, phenols, ethers, aldehydes, ketones, steroids, lactones, and so on. The predicted heating values exhibit high consistency with the theoretical values and small standard errors, thereby confirming the reliability of estimated combustion heat using bond dissociation energies.

It is widely recognized that the bond dissociation energy (BDE) values increase proportionally with the difference in electronegativities of bonded atoms, as reported by Blanksby and Ellison [19] as well as Tiggelen et al. [21]. Based on the findings presented in Figure 1, disregarding the influence of chemical groups on BDE values has an insignificant impact on the combustion heats of organic substances. This also confirms the validity of simplifying a chemical bond as either a single bond or combination of single bonds when calculating the combustion heat of organic matters.

**Figure 2:**
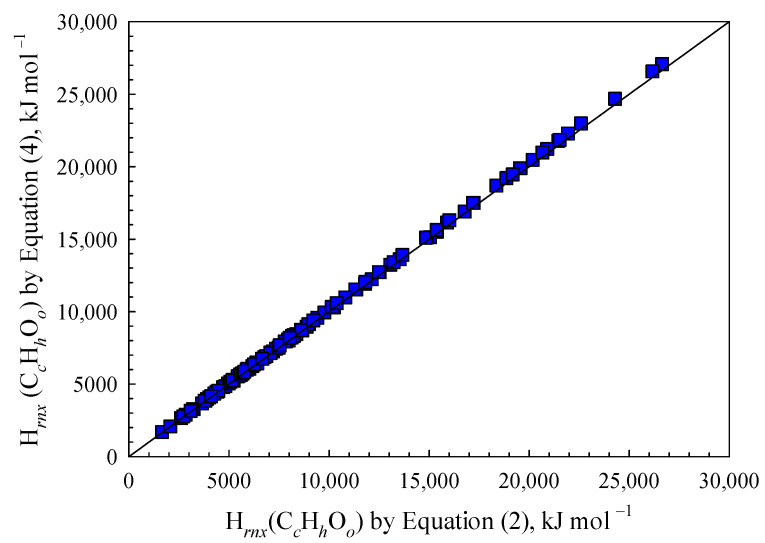
Comparison of H*_rnx_*(C*_c_*H*_h_*O*_o_*) values determined using Equations (2) and (4).

Moreover, the results illustrated in Figure 1 indirectly support the effectiveness of the empirical formula Equation (4) for estimating BDE values and combustion heats. This is further substantiated by the findings presented in Figure 2. A comparison was conducted between the total bond dissociation energies (BDEs) predicted using Equation (4) and those estimated using Equation (2) for 154 species of organic compounds. Negligible errors were observed, indicating that the empirical Formula (4) performs exceptionally well in estimating H*_rxn_*(C*_c_*H*_h_*O*_o_*) of organic materials.

## 4. Results and Discussion

**Figure 3:**
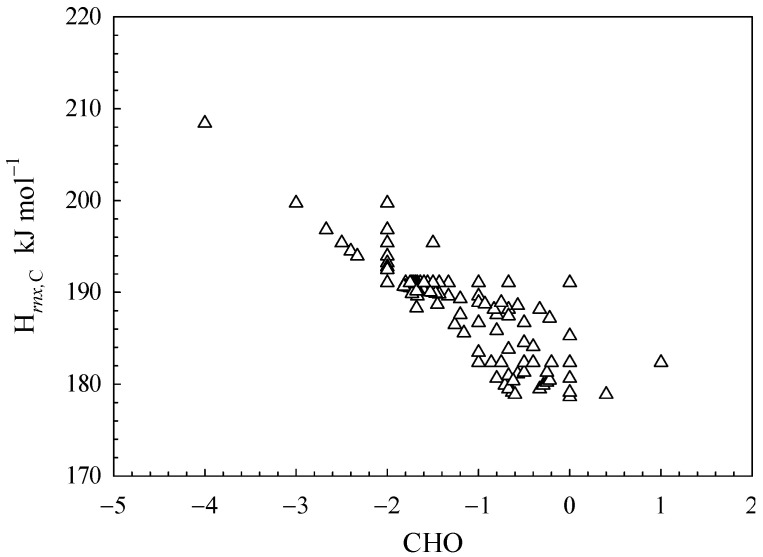
The correlation between H*_rnx_*_,C_ values and CHO values (CHO = (2·*o*-*h*)/*c*).

The parameters H*_rnx,_*_C_, H*_rnx,_*_H_, and H*_rnx,_*_O_ in Equation (5) correspond to the contributions of individual elemental atoms toward the calculated bond dissociation energy values of an organic compound. These parameters are contingent upon the chemical structure of the compound. For instance, for carbon atoms in methane (CH_4_), the value of α_C,*m*_ is consistently equal to 1, thereby resulting in an H*_rnx_*_,C_ value of 208.42 kJ mol^−1^. In contrast, the carbon bond in methanol (CH_3_OH) comprises three C-H bonds and one C-O bond, thereby enabling the determination of the H*_rnx_*_,C_ value as 199.73 kJ mol^−1^. As depicted in Figure 3, the H*_rnx_*_,C_ value varies in the range of 178.63 to 208.42 kJ mol^−1^.

As indicated by Equations (4) and (5), the H*_rnx_*_,C_ value increases with a decrease in average α_C,*m*_ value. As per Equation (3), the value of α_C,*m*_ can be either 1.2 or 1, resulting in a theoretical range for H*_rnx_*,_C_ of 173.68 to 208.42 kJ mol^−1^. The maximum H*_rnx_*,_C_ value is equivalent to that of CH_4_, as depicted in Figure 3. However, the predicted minimum value is slightly lower than the experimental values, and no actual organic compound exhibits it. This is due to the indispensability of C-H bonds in the organic matter’s C bonds.

The CHO index, as depicted in Figure 3, has been employed to characterize the status of carbon bonds. The H*_rnx,C_* values display an inverse correlation with increasing CHO values. A higher value of the CHO index indicates a greater degree of carbon oxidation in organic matter, leading to a reduction in the proportion of C-H bonds within organic compounds’ C bonds. This results in the higher values of the average α_C,*m*_ of organic materials, due to the higher min(α_C_, α_O_) value compared to the min(α_C_, α_H_) value. In view of Equation (4), it is inevitable that the H*_rnx,C_* value will decrease. From a chemical mechanism perspective, the breaking of C-O bonds is more facile than that of C-H bonds.

**Figure 4:**
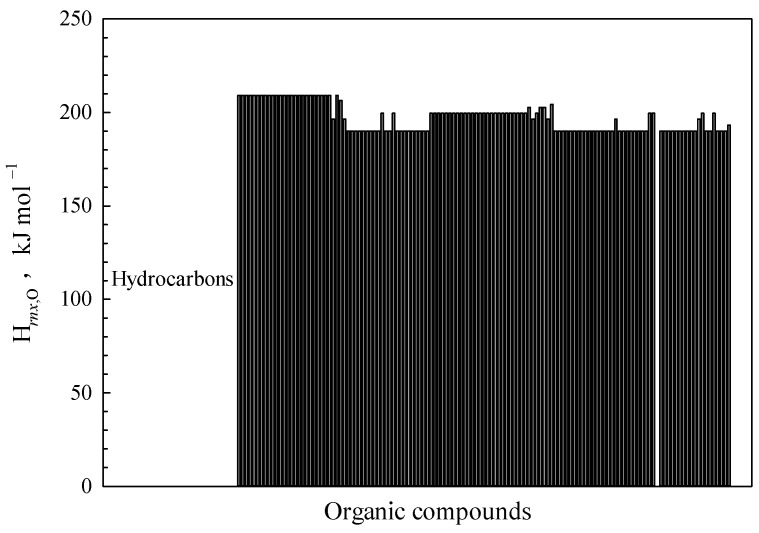
Variation trend of the values of H*_rnx_*_,O_ for 154 species of organic compounds.

The experimental H*_rnx,_*_O_ values demonstrate a limited range of fluctuation, ranging from 190.07 to 209.09 kJ mol^−1^ across a set of 154 organic compounds, as depicted in Figure 4. The observed results align with the theoretical predictions, demonstrating consistency between theory and experiment. As the data samples do not contain any O-O bonds, the α_O,*m*_ value would be 1.2 or 1. When all oxygen bonds are either C-O or C=O bonds, the theoretical lower limit value is found to be consistent with the experimental data, i.e., α_O_*D*_O_/α_C_.

The α_O,*m*_ value, however, would not be uniformly equal to 1 since the oxygen bonds in organic matters are not exclusively composed of O-H bonds. Theoretical maximum values of H*_rnx,_*_O_ can be deduced when an oxygen atom is bonded to both a hydrogen and a carbon, suggesting that alcohols are the most likely candidates in this group. Then, the upper limit value of H*_rnx_*_,O_ can be expressed as $\frac{\alpha_{O}D_{O}}{2}\left(\frac{1}{\alpha_{C}}+\frac{1}{\alpha_{H}}\right)$, where the calculated results align with the statistical findings from the experiments.

According to the definition of parameter *α* [21], the value of α for any radical is equal to or greater than unity. Therefore, irrespective of the nature of the hydrogen bond, the α_H,*m*_ value will consistently remain equal to 1. Consequently, this leads to H*_rnx,_*_H_ = *D*_H_. Thus, the value of H*_rnx,_*_H_ remains a constant of 215.95 kJ mol^−1^.

**Figure 5:**
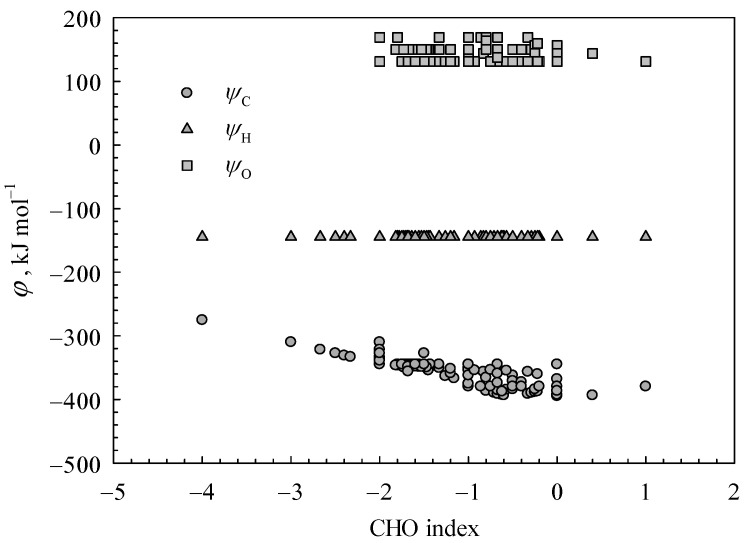
The correlation between coefficients *φ*_C_, *φ*_H,_ and *φ*_O_ and CHO index.

The variation trends of coefficients *φ*_C_, *φ*_H,_ and *φ*_O_, along with the CHO values, are illustrated in Figure 5. In accordance with the expression of Equation (6), the parameters *φ*_C_, *φ*_H,_ and *φ*_O_ characterize the energy output per mole of an elemental atom in relation to the higher heating values of organic substances. The *φ*_H_ value has been determined as a constant of −144.44 kJ mol^−1^, as H*_rnx,_*_O2_ and H*_rnx,_*_H2O_ in Equation (8) are constants. Based on the empirical model presented in Equation (3), it is deduced that *D*_H_ ≈ 0.5·H*_rnx_* (H_2_). Considering Equation (8) and the hydrogen combustion reaction, it can be inferred that *φ*_H_ is approximately equal to 0.5·HHV*_m_*(H_2_). The combustion heat of H_2_ is approximately −142.30 kJ mol^−1^ [5], which closely aligns with the *φ*_H_.

As depicted in Figure 5, the parameter *φ*_C_ is observed to range from −394.50 to −275.34 kJ mol^−1^ and exhibits a significant decrease with an increase in CHO index. The higher CHO index value indicates a reduced number of carbon atoms participating in the oxidation reaction during the combustion process. Accordingly, the contribution of carbon to the calorific value of combustion is reduced.

In consideration of the theoretical range (173.68, 208.42] kJ mol^−1^ for parameter H*_rnx_*_,C_, it is theoretically anticipated that the coefficient *φ*_C_ would fall within the range (−414.30, −275.34] kJ mol^−1^. The decrease in energy released via carbon oxidation is remarkable as the H*_rnx_*_,C_ value increases. Therefore, achieving the minimum of *φ*_C_ is theoretically impossible.

Assuming all carbon bonds are C-C bonds, we can calculate that H*_rnx,_*_C_ = *D_C_*. Then, the estimated value of *φ*_C_ can be determined as −414.30 kJ mol^−1^, slightly exceeding the heat of combustion for elemental carbon (−393.5 kJ mol^−1^). The observed phenomenon is consistent with the fact that C-C bonds in carbon substances exhibit greater stability compared to those present in organic materials. The actual value of *φ*_C_ is typically significantly higher than the lower limit value, owing to the fact that the cleavage of C-H bonds requires a greater amount of energy absorption in comparison to C-C bonds.

According to Equation (9), the theoretical value of *φ*_O_ would be between 131.13 and 169.17 kJ mol^−1^, in light of the theoretical range [190.07, 209.09] kJ mol^−1^ of the H*_rnx_*_,O_ value. This is consistent with the statistical results as shown in Figure 5. Notably, Equation (9) denotes the difference in bond dissociation energy between O bonds in organic compounds and those in molecular oxygen O_2_.

**Figure 6:**
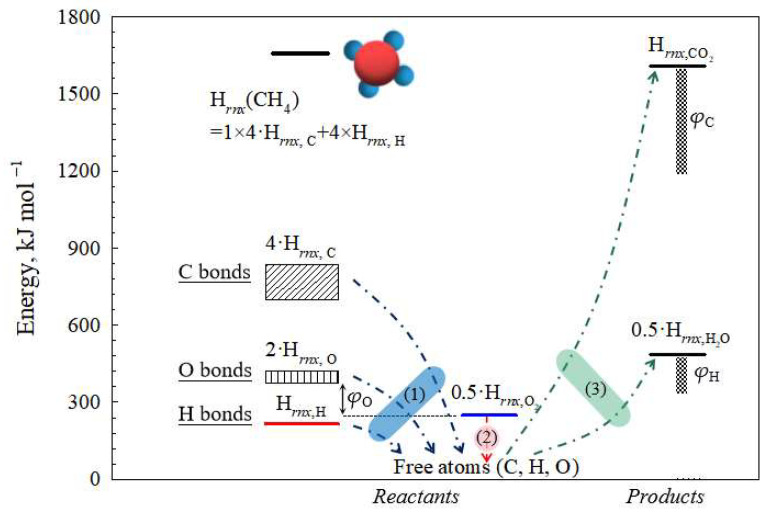
The proposed reaction paths of combustion process for organic materials.

Based on the aforementioned analysis and Equations (6)–(9), the combustion process of organic compounds can be delineated into three distinct steps as shown in Figure 6: (1) Thermal decomposition of organics yields isolated carbon, hydrogen, and oxygen atoms. (2) The molecular oxygen O_2_ produces the free atom O. (3) The carbon and hydrogen atoms directly combine with oxygen atoms to produce carbon dioxide and water. The energy required for the formation of free O atoms in organic compounds is evidently higher compared to that needed for molecular oxygen.

The phenomenon of *φ*_O_ > 0 demonstrates that the presence of oxygen in organic compounds has a negative impact on their combustion heat, which is consistent with statistical findings documented in the previous scholarly literature [4,5,8,23]. However, the current mathematical fittings occasionally adhere to the fundamental chemical principle regarding the role of element O in combustion heat.

By analyzing a significant amount of experimental data, previous research suggests that the heat released from burning organic fuels is approximately 418 kJ per mole of oxygen consumed, with water generated in the gas phase [24,25]. This empirical theory is commonly known as the oxygen consumption method [4,22]. By performing linear regression analysis, we have obtained HHV*_m_* ≈ −437.81·*ν*_O2_ with a high *R*^2^ value of 0.9988 for 154 different species of organic compounds. This can be further substantiated through theoretical deduction based on the proposed theoretical Equations (6)–(9). By utilizing the aforementioned hypothesis and Equation (3), Equation (6) can be rewritten as follows:

(10) $$\mathrm{HHV}m\approx \left(H_{rnx,\;O_{2}}-4\alpha_{O}D_{O}\right)\cdot \nu_{O_{2}}$$

By substituting the given values, the coefficient (H*_rnx_*_,O2_ − 4α_O_*D*_O_) can be computed as −414.32 kJ mol^−1^. Equation (10) suggests that the heat released during the combustion process mainly depends on the bond recombination of atomic oxygen in oxygen molecules, regardless of changes in bond dissociation energies for atomic carbon, hydrogen, and oxygen in organic matter before and after reaction. This elegantly clarifies the correlation between combustion calorific value and oxygen consumption in terms of bond dissociation energy, providing a clear perspective.

For high-molecular polymers with complex constituents, the uncertainty surrounding their chemical structures hinders the applicability of Equation (2) in predicting H*_rnx,i_* and HHV*_m_* values. Consequently, empirical correlations are frequently established based on mass fractions of elemental composition. For high-molecular polymers with the chemical formula C*_c_*H*_h_*O*_o_*, we define [C], [H], and [O] as the mass fractions of carbon, hydrogen, and oxygen elements, respectively. Then, we have [C] = 12*c*/M*_s_*, [H] = *h*/M*_s_*, and [O] = 16*o*/M*_s_*, where M*_s_ =* (12*c* + *h*+16*o*). The higher heating values on a mass basis HHV*_g_* can be expressed as HHV*_m_*/M*_s_*. On the basis, Equation (6) can be rewritten as

(11) $${\mathrm{HHV}}_{g}\approx \frac{\varphi_{C}}{12}\cdot \left[C\right]+\varphi_{H}\cdot [H]+\frac{\varphi_{O}}{16}\cdot [O]$$

By employing a genetic algorithm optimization methodology in the 1stOpt 10.0 software, an analytical model is developed using data from a set of 154 organic matters [22] while adhering to specific constraint conditions *φ*_C_∈(−414.30, −275.34], *φ*_H_ = −144.44, and *φ*_O_∈[131.13, 169.17]. The fitting result has been listed as Equation (12) in Table 2, demonstrating a significantly high correlation coefficient of *R*^2^ = 0.9969.

**Figure 7:**
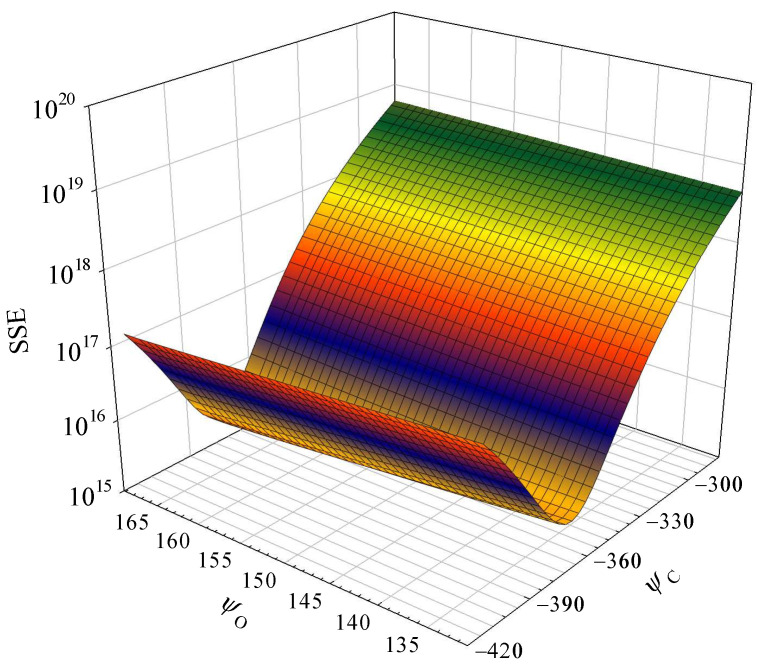
Parameter sensitivity analysis of the theoretical model Equation (12).

The sum of squares due to error (SSE) of parameters in Equation (12) is illustrated in Figure 7. The SSE exhibits significant variations in response to changes in parameter *φ*_C_. The sensitivity values of *φ*_C_ and *φ*_O_ are calculated as 92.65% and 7.35%, respectively. Consequently, prior research has occasionally established a correlation between the higher heating value and carbon fractions using the equation HHV_g_ = *a*·[C] + *b* in the literature [25,26,27]. The correlation for the organic compounds in the present work is listed as Equation (13) in Table 2. The correlation coefficient exhibits a strong positive relationship with a value of 0.96.

Table 2 also presents other established correlations that share the same structure as Equation (11). The theoretical coefficients in Equation (11) prior to the mass fractions [C], [H], and [O] can be calculated as (−34.53, −22.95], −144.44, and [8.20, 10.57], respectively. The coefficients associated with [C] in Equations (14)–(18) consistently align with the expected theoretical range, while those connected to [H] exhibit noticeable variations. However, the observed empirical correlations sometimes challenge the established role of oxygen in the combustion process of organic substances, as demonstrated using Equations (14) and (15).

**Figure 8:**
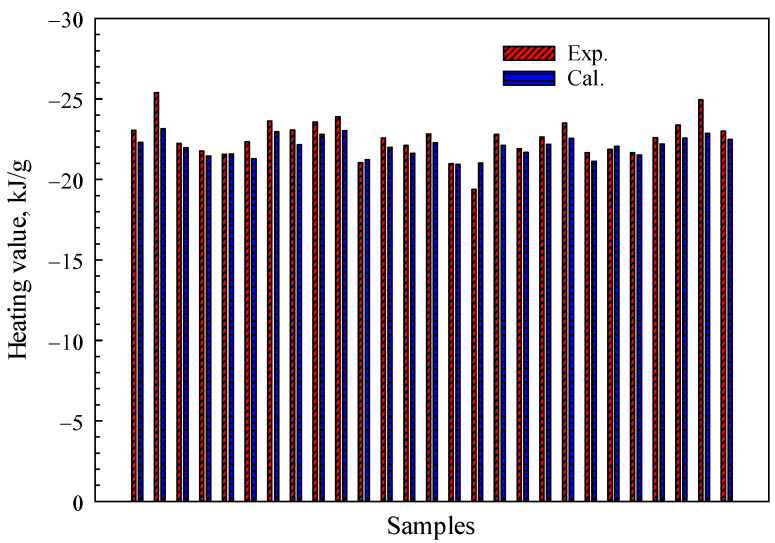
The comparison between the heating values calculated using Equation (12) and the experimental data.

As shown in Figure 8, to further investigate the robustness of the proposed theoretical models, we have compared the calculated results obtained from Equation (12) with experimental data collected from 27 different species of woody plant foliage [18]. The test data adopted here are on a dry, ash-free basis. The average relative error, as depicted in Figure 8, amounts to approximately 2%. The distinction arises from the fact that Equation (12) employs organic matter without benzene rings, whereas biomass primarily consists of cellulose, hemicellulose, and lignin, which contain a significant number of benzene rings. Theoretical models for Equations (6)–(9) do not take into account nitrogen and sulfur, which are other potential sources of error that should be considered.

For polymer materials with the chemical formula C*_c_*H*_h_*O*_o_*N*_n_*S*_s_*, the combustion process can be expressed as

(21) $$C_{c}H_{h}O_{o}N_{n}S_{s}+\nu_{O_{2}}O_{2}\;\to \;\nu_{CO_{2}}CO_{2}+\;\nu_{H_{2}O}H_{2}O+\nu_{N_{2}}N_{2}+\;\nu_{SO_{2}}SO_{2}$$

The heat of combustion of reaction (21) can be expressed using the extension Equation (6) according to the proposed theoretical model.

(22) $${\mathrm{HHV}}_{g}\approx \frac{\varphi_{C}}{12}\cdot \left[C\right]+\varphi_{H}\cdot [H]+\frac{\varphi_{O}}{16}\cdot [O]+\frac{\varphi_{N}}{14}\cdot [N]+\frac{\varphi_{S}}{32}\cdot [S]$$

where *φ*_N_ = 3·H*_rnx_*_,N_ − 0.5·H*_rnx_*_,N2_, *φ*_S_ = 4·H*_rnx_*_,S_ − H*_rnx_*_,SO2_, and the values of H*_rnx_*_,N2_ and H*_rnx_*_,SO2_ are equivalent to 946 kJ mol^−1^ and 1072.36 kJ mol^−1^, respectively. The maximum values of H*_rnx_*_,N_ and H*_rnx_*_,S_ can be determined as α_N_D_N_ and α_S_D_S_. The bond dissociation energies (BDE) of the N-H bond and S-H are reported as 390.80 kJ mol^−1^ and 339.00 kJ mol^−1^, respectively [19,20]. According to Equation (3), the values of H*_rnx_*_,N_ and H*_rnx_*_,S_ should be below 174.85 kJ mol^−1^ and 123.05 kJ mol^−1^, respectively. Subsequently, due to the aforementioned constrained condition, Equation (22) can be effectively fitted using a genetic algorithm in the 1stOpt 10.0 software as

HHV*_g_* = −33.71·[C]−144.44·[H] + 12.62·[O] + 3.68·[N]−18.13·[S]  *R*^2^ = 0.9505 (23)

**Figure 9:**
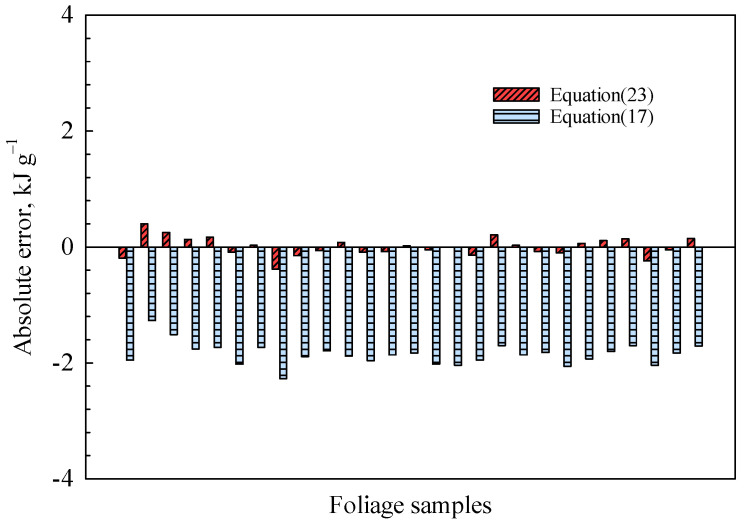
The absolute error of HHV prediction model proposed by present author and Dulong.

**Figure 10:**
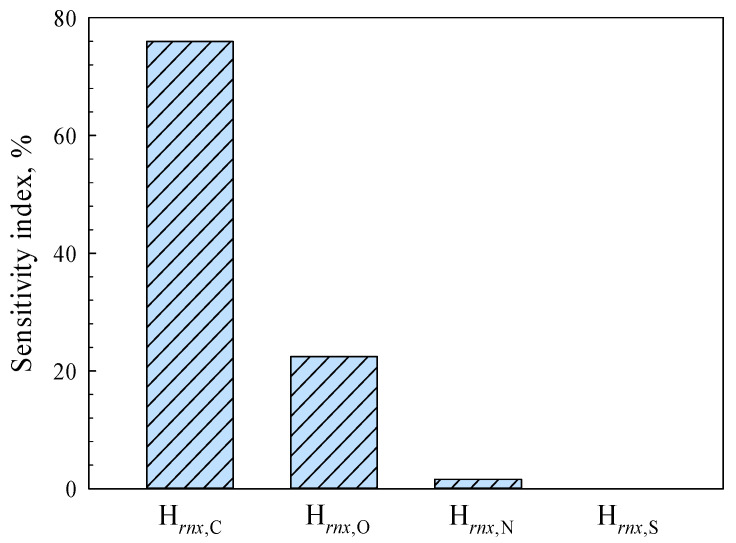
The sensitivity indices of parameters H*_rnx_*_,C_, H*_rnx_*_,O_, H*_rnx_*_,N_, and H*_rnx_*_,S_ for Equation (23).

The present correlation, based on the theoretical model, evidently aligns with the mathematical fitting outcomes as listed in Table 2. The absolute error between the experimental data and the results predicted with Equations (19) and (23) is depicted in Figure 9. Equation (19) was initially proposed by Dulong and has since gained widespread utilization across various disciplines [24]. The predictive model proposed in the present study clearly outperforms the existing model. The proposed model incorporates the influence of the element N, and the inclusion of conditional constraints enhances the rationality of coefficients. The influence of element contents on the accuracy of predicted results, however, necessitates further discussion. Hence, we have examined the parameter sensitivity of Equation (23) as illustrated in Figure 10.

As shown in Figure 10, the fitting parameters H*_rnx_*_,N_ and H*_rnx_*_,S_ have really small sensitivity index values, which means it is okay to ignore the mass fractions of element N and element S when predicting the higher heating values of lignocellulosic materials. This finding aligns with the comparative outcome illustrated in Figure 9. The results of parameter sensitivity analysis critically depend on the numerical distribution of variables, which should be noted. Lignocellulosic biomass are essentially high-molecular polymers, mainly involving cellulose, hemicellulose, and lignin. According to the statistical data, lignocellulosic materials exhibit the highest carbon (C) content, followed by oxygen (O). The hydrogen (H) content is comparatively lower, while nitrogen (N) and sulfur (S) elements are present minimally or negligibly [28]. Therefore, Figure 10 implies that there is a potential risk of over-fitting when establishing empirical correlations involving the mass fractions of elements N and S in lignocellulosic materials. The efficacy of Equation (11) in predicting the performance of biomass materials and municipal solid wastes can be attributed to this observation.

## 5. Conclusions

Based on bond dissociation energies (BDE), we have developed a theoretical model for estimating the higher heating values of organic compounds, which has been proven reliable with theoretical data from typical organic materials. Our analysis indicates that the energy contribution of one mole of hydrogen atoms is approximately half the combustion heat of H_2_ (−144.4 kJ mol^−1^). Further investigation reveals significant variations in the bond dissociation energies (BDEs) of carbon atoms within organic compounds, resulting in a range of energy outputs from −414.30 to −275.34 kJ mol^−1^ per mole of carbon atoms. The presence of oxygen atoms in organic compounds has a negative impact on the magnitude of combustion heat, with values ranging from 131.1 to 207.17 kJ mol^−1^.

By adhering to the constraints of the combustion mechanism, this study presents a novel model: HHV*_g_* = −31.34·[C] − 144.44·[H] + 10.57·[O]. Based on the parameter sensitivity analysis, the coefficient associated with carbon mass fraction exhibits a significantly greater impact on the prediction accuracy of results, demonstrating a sensitivity value of 92.65%. Further analysis suggests that empirical correlations involving the mass fractions of elements N and S in lignocellulosic materials may be susceptible to over-fitting.

## Figures and Tables

**Table 1:** Average bond dissociation energies at 298 K (kJ mol^−1^) [19,20].

Bond	H-H	C-C	O-O	C=C	C≡C	O=O *^a^*	H-C	H-O	C-O	C=O	C=O *^b^*
kcal/mol	104.2	83.0	35.0	146.0	210.0	119.0	99.0	111.0	85.0	178.0	192.0
kJ/mol	436.1	347.4	146.5	611.0	878.9	498.0	414.3	464.5	355.7	744.9	803.5

*^a^* The exact value of O=O bond enthalpy in O_2._ *^b^* The exact value of C=O bond enthalpy in CO_2._

**Table 2:** Typical correlations proposed for combustion heat (kJ g^−1^) of organic compounds.

No.	Authors	Years	Correlation
Equation (12)	Present study	2023	HHV*_g_* = −31.34·[C] − 144.44·[H] + 10.57·[O]
Equation (13)	Present study	2023	HHV*_g_* = −34.52·[C] − 9.09
Equation (14)	Jenkins and Ebeling [14]	1985	HHV*_g_* = −29.34·[C] − 51.74·[H] − 5.64·[O]
Equation (15)	Sheng and Azevedo [13]	2005	HHV*_g_* = −30.00·[C] − 68.72·[H] − 1.81·[O]
Equation (16)	Schmidt-Rohr [4]	2015	HHV*_g_* = −34.83·[C] − 125.40·[H] + 13.06·[O]
Equation (17)	Tao et al. [23]	2016	HHV*_g_* = −30.99·[C] − 149.99·[H] + 10.22·[O]
Equation (18)	Huang and Luo [8]	2020	HHV*_g_* = −34.47·[C] − 119.20·[H] + 11.30·[O]
Equation (19)	Dulong [24]	1945	HHV*_g_* = −33.91·[C] − 144.44·[H] + 18.05·[O] − 9.42·[S]
Equation (20)	Huang and Luo [8]	2000	HHV*_g_* = −34.43·[C] − 119.20·[H] + 11.30·[O] + 2.40·[N] − 9.30·[S]

## Data Availability

The data presented in this study are available on request from the corresponding author. The data are not publicly available due to privacy.
